# What could this dark lesion on a woman’s eyelid be?

**DOI:** 10.1097/JW9.0000000000000037

**Published:** 2022-08-24

**Authors:** Maria Victoria Rosso, Maria Victoria Rodriguez Kowalczuk, Maria Manuela Martinez Piva, Luis Daniel Mazzuoccolo

**Affiliations:** a Dermatology Department, Hospital Italiano de Buenos Aires, Buenos Aires, Argentina; b Head of Dermatology Department, Hospital Italiano de Buenos Aires, Buenos Aires, Argentina

**Question 1**

**What is your diagnosis?**

A. Blue nevus
B. Melanoma
C. Trichoblastoma
D. Pigmented apocrine hidrocystoma
E. Pigmented basal cell carcinoma

**Correct answer:** D. Pigmented apocrine hidrocystoma. Apocrine hidrocystomas (AHs) are rare, benign, cystic lesions of apocrine glands. A pigmented variant has been described, characterized by a darker coloration related to lipofuscin pigments, hemosiderin, melanin deposits, or Tyndall effect.

## Case summary

**Fig. 1.**
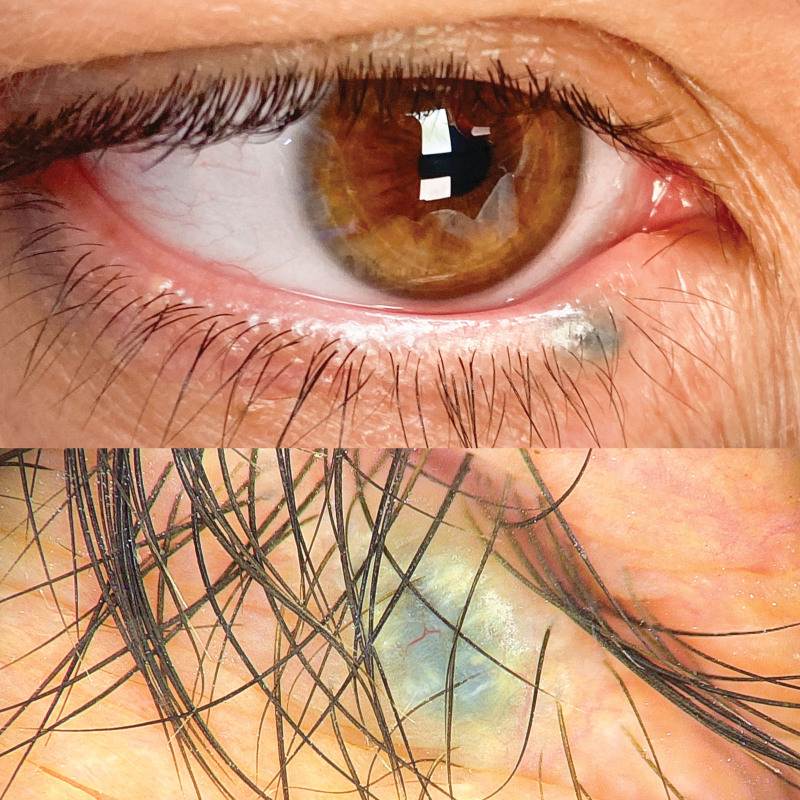
Physical examination and dermoscopy. (Top) Right inferior eyelid well-defined hyperpigmented tumor. (Bottom) Dermoscopy revealing a blue-gray area with peripheral shiny white streaks and an arborizing central vessel.

A 42-year-old woman (Fitzpatrick type IV) presented with a 4-year history of hyperpigmented growing asymptomatic lesion on her right inferior eyelid. Physical exam revealed a dark bluish 3 mm tumor and dermoscopy showed a blue-gray area with peripheral shiny white streaks and an arborizing central vessel (Fig. 1). Excisional biopsy was performed and on gross examination and dissection, it drained a dark brown granular liquid. Histopathology was consistent with a pigmented apocrine hidrocystoma.

**Question 2**

**What are you most likely to see on pathology?**

A. Excess mature apocrine glands in the reticular dermis, extending into the subcutaneous fat.
B. A dermal cyst with a cavity partially replaced by a papillary or adenomatous proliferation.
C. An intradermal cyst lined by a low cuboidal or flattened epithelium with paler cytoplasm and a watery secretion without cytoplasmic contributions.
D. An intradermal cyst lined by an outer layer of flattened vacuolated cells and an inner layer of tall columnar cells with eosinophilic cytoplasm, vesicular nuclei with secretory decapitation and periodic acid-Schiff (PAS)-positive granules.
E. Villi covered by a double-layered epithelium consisting of an inner zone of small cells and an outer zone of tall columnar cells, without cytologic atypia.

**Correct answer:** D. An intradermal cyst lined by an outer layer of flattened vacuolated cells and an inner layer of tall columnar cells with eosinophilic cytoplasm, vesicular nuclei with secretory decapitation and PAS-positive granules. Decapitation secretion is usually present, and it is typical of apocrine glands. Pigmented variants contain lipofuscin, hemosiderin, or melanin deposits in a colloidal suspension that can be revealed by different stains including PAS with diastase, Sudan Black, or Fite acid-fast.

**Question 3**

**Which is the most common dermoscopic feature of apocrine hidrocystomas?**

A. A homogeneous area that occupies the whole lesion
B. Arborizing vessels
C. Multiple gray-blue globules
D. Shiny white streaks
E. Blue-white veil

**Correct answer:** A. A homogeneous area that occupies the whole lesion. Most commonly, on dermoscopy, AHs show a skin-colored, yellow, or blue homogeneous area that occupies the whole lesion. The second most frequent feature is arborizing vessels, followed by shiny white streaks.

## Discussion

Eccrine and AHs are the most common adnexal eyelid tumors. They are considered nonproliferative retention cysts.^1^ AHs are usually found on the head and neck affecting the eyelids and cheeks most commonly.^2^

Eyelid hidrocystomas typically affect older patients without gender distinction unless there are multiple cysts, where women outnumber men.^1,3^ Classically, eccrine hidrocystomas are described as more frequent but recent studies have shown that apocrine cysts predominate.^3^

The clinical presentation is an asymptomatic, 1 to 4 mm, skin dome-shaped cystic papule or nodule on the lower eyelid with a predominance for the left eye. The lesions can be skin colored, translucent, milky white, blue, amber, brown, or black in coloration.^1^ The reasons for the more frequent involvement of the lower eyelid and the higher incidence of left side cysts remain unknown. AHs rarely cause madarosis as opposed to malignant tumors. The former do not affect vision unless they are multiple and/or develop a considerable size. On palpation, hidrocystomas are soft and compressible.^3^ The pigmented variants might be misdiagnosed as vascular, pigmented epithelial, or melanocytic proliferations such as angiokeratoma, seborrheic keratosis, blue nevus, and melanoma.^1^

Chromhidrosis describes the excretion of colored secretions composed of lipofuscin pigments in apocrine gland-rich anatomic locations. The darker coloration is believed to be caused by the Tyndall phenomenon. Hemosiderin or melanin deposits are rarely identified in hidrocystomas. Nonetheless, the Tyndall effect rather than specific pigments is thought to be the source of the color we perceive in clinically pigmented hidrocystomas.^1,3^

Histologically, they are large unilocular or multilocular dermal cysts typically lined by a bilayer of epithelial cells with an outer layer consisting of myoepithelial cells and an inner layer of tall columnar cells. Decapitation secretion is usually present and is the hallmark of apocrine glands.^2^. Lipofuscins can be detected by fluorescent microscopy and histochemistry for PAS with diastase, Sudan Black or Fite acid-fast.^1^

A recent multicenter study analyzed the dermoscopic features of 22 cases of AHs with the most frequent findings being: a homogeneous area that occupies the whole lesion (100%), arborizing vessels (68.2%), and shiny white streaks (22.7%). The commonest detected colors were: skin colored, yellow, and less frequently blue.^2^

There are multiple therapeutic modalities to remove these lesions such as excision, injection of trichloroacetic acid, botulinum toxin (blocks cholinergic terminals of apocrine glands, diminishing its secretion), topical application of atropine (an anticholinergic agent, reduces glands release), electrodesiccation, and laser therapy. However, when hidrocystomas are pigmented or exhibit concerning features leading to suspicion of malignancy, histopathologic confirmation is recommended.^2,3^

Dermatologists must be aware of pigmented hidrocystomas because although being rare, they mimic cutaneous malignancies. In order to improve the diagnostic accuracy, we report the clinical and dermoscopic aspects of these benign palpebral tumors.

## Conflicts of interest

None.

## Funding

None.

## Author contributions

MVR participated in drafting the article, data collection, conception or design of the work, critical revision of the article, and final approval of the version to be published. MVRK participated in data collection, conception or design of the work, critical revision of the article, and final approval of the version to be published. MMMP and LDM participated in conception or design of the work, critical revision of the article, and final approval of the version to be published.

## Study approval

The author(s) confirm that any aspect of the work covered in this manuscript that has involved human patients has been conducted with the ethical approval of all relevant bodies.

## Patient consent

Patient consent has been obtained to publish these photographs and case report.
